# The effects of physical activity on social-emotional competence in primary school students: a meta-analysis

**DOI:** 10.3389/fpsyg.2025.1657165

**Published:** 2025-11-13

**Authors:** Simei Fu, Pengfei Wen, Jinsong Wu, Zhi Li, Yuan Zheng

**Affiliations:** 1 School of Physical Education, Guangzhou Sport University, Guangzhou, Guangdong, China; 2 School of Physical Education and Sports Science, South China Normal University, Guangzhou, Guangdong, China

**Keywords:** social-emotional competence, primary school students, physical activity, meta-analysis, intervention

## Abstract

**Introduction:**

Children and adolescents globally face escalating mental health challenges, with approximately one in seven experiencing mental health conditions. This phenomenon is closely linked to underdeveloped social-emotional competence (SEC), highlighting the urgency of prioritizing and strengthening socioemotional development. The primary school years represent a developmentally sensitive period for cultivating SEC, making this stage particularly crucial. As a viable intervention approach, physical activity (PA) demonstrates established associations with multiple dimensions of SEC. Consequently, this meta-analysis systematically integrates and quantitatively evaluates empirical studies involving primary school students aged 5-13 years to determine the overall effect of PA interventions on SEC.

**Methods:**

This review strictly followed the Preferred Reporting Items for Systematic Reviews and Meta-Analyses (PRISMA) guidelines and the Practical Guide for Transparent Reporting of Systematic Reviews. We included randomized controlled trials (RCTs) and cluster randomized controlled trials (cRCTs) examining PA interventions targeting SEC in primary school students. Cohen's d served as our primary effect size metric.

**Results:**

Twelve studies were included in this meta-analysis. The results indicated that PA intervention improved SEC in primary school students [*d* = 0.37, 95% *CI* (0.20, 0.55), *p* < 0.001]. Moderator analysis revealed that structured PA and shorter-length interventions ( ≤ 12 weeks) yielded superior effects on SEC. Furthermore, the effect size was significantly higher for special needs children [children with Autism Spectrum Disorder (ASD) and Attention-deficit hyperactivity disorder (ADHD)] compared to typically developing children. Pre-pandemic physical activity interventions demonstrated significantly more positive effects on SEC than those implemented post-pandemic.

**Discussion:**

The findings of this meta-analysis provide robust evidence for the effectiveness of PA interventions in enhancing SEC among primary school students. Moderator analyses indicate that the effect is moderated by PA type, participant group, Length of Intervention, and Research Period. These findings underscore the significant potential of PA as a feasible solution for improving SEC in this population.

**Systematic review registration:**

https://www.crd.york.ac.uk/PROSPERO/view/CRD420251012368, identifier: CRD420251012368.

## 1 Introduction

In recent years, cultivating students' social-emotional competence (SEC) has increasingly become a core issue in the global education field. The Education 2030 Framework for Action published by United Nations Educational, Scientific and Cultural Organization (UNESCO) emphasizes the need to prioritize the cultivation of children's SEC (Mundial and UNICEF, 2016). The OECD Learning Framework 2030 issued by the Organization for Economic Co-operation and Development (OECD) proposes that SEC is regarded as one of the key skills for responding to future changes and has also become an important component in the global evaluation and monitoring of children's and adolescents' abilities. Developed countries worldwide have already established fostering SEC as a crucial objective of school education (Huang, 2022).

SEC is a multidimensional set of skills, defined as the process encompassing an individual's core abilities to acquire, recognize, and manage emotions; set and achieve positive goals; understand others' perspectives; establish and maintain positive relationships; make responsible decisions; and constructively handle interpersonal situations (Elias et al., 1997). In the report Skills for Social Progress: The Power of Social and Emotional Skills, the OECD, based on the “Big Five Model of Personality”, divides SEC into five core competencies (task performance, emotional regulation, collaboration, open-mindedness, and engaging with others). Each competency is further defined by three sub-competencies: task performance encompasses self-control, responsibility, and perseverance; emotional regulation includes stress resistance, optimism, and emotional control; collaboration involves empathy, cooperation, and trust; open-mindedness contains curiosity, creativity, and tolerance; engaging with others is reflected in energy, assertiveness, and sociability. These collectively capturing the multifaceted proficiencies essential for individual effectiveness in social and emotional domains (Yuan et al., 2021; Organization for Economic Co-Operation and Development (OECD), 2015).

Mental health issues among children and adolescents are increasingly severe globally. A joint report issued by the World Health Organization (WHO) and the United Nations Children's Fund (UNICEF) reveals that roughly one in seven children and adolescents worldwide experience mental health conditions. Approximately 8% of children aged 5-9 years and about 14% of adolescents aged 10-19 years are affected by mental disorders. These include anxiety disorders, depression, Attention-deficit hyperactivity disorder (ADHD), and conduct disorder, among others [(UNICEF), 2024]. These problems seriously harm the physical and mental health of children and adolescents and reduce their academic engagement and social participation (Ghandour et al., 2019). The emergence of such mental health issues is closely linked to insufficient development of SEC, highlighting the urgency of focusing on and strengthening SEC cultivation. The primary school years represent a critical period for SEC development (Zhang, 2022), holding particular importance. Research confirms that developing SEC during this period yields significant benefits for primary school students, not only aiding in the prevention and alleviation of psychological problems (Durlak et al., 2011; Greenberg et al., 2003), but also significantly enhancing academic engagement and learning outcomes, as well as promoting the establishment and maintenance of positive peer relationships (Currie et al., 2016; Wigelsworth et al., 2020).

Currently, Physical activity (PA) is receiving widespread attention in the fields of education and mental health as a significant intervention for enhancing SEC. A substantial body of empirical research indicates that PA not only offers significant physiological benefits, but also provides children with practical settings for collaborative interaction, understanding of rules, emotional regulation, conflict resolution, and goal setting–key components of SEC (Ahn and Fedewa, 2011; Dale et al., 2019). Specifically, diverse forms of PA such as Judo (Ludyga et al., 2022), Taekwondo (Roh et al., 2018), physical games (Luna et al., 2021; Zhou, 2023), dance (Bollimbala et al., 2019), aerobic exercise (Tse, 2020; Williams et al., 2019), physical training (Haghighi et al., 2023), and structured physical activity (Condello et al., 2021; Ángel Latorre-Román et al., 2021; Najafabadi et al., 2018; Zhao and Chen, 2018a) have been shown to positively impact multiple dimensions of SEC in primary school students. However, existing studies exhibit significant heterogeneity in participant demographics (e.g., age, gender, cultural background), intervention parameters (e.g., activity type, frequency, duration), and the dimensions of SEC measured. This heterogeneity has led to considerable variation in research findings, making it difficult to draw definitive conclusions regarding the overall effectiveness of PA interventions or the differential impacts of various PA types and implementation parameters. Based on this, the present study aims to systematically integrate and quantitatively assess relevant research involving primary school students aged 5–13 years using meta-analytic methods, seeking to clarify the overall intervention effect of PA on SEC and elucidate its specific impacts on the core sub-domains of SEC (namely task performance, emotional regulation, collaboration, open-mindedness, and engaging with others). Concurrently, this study will explore potential moderating factors, including intervention duration, sample characteristics, and type of PA, striving to uncover the key mechanisms influencing intervention effectiveness. Through the above analyses, this study aims to provide systematic and robust empirical evidence for PA intervention programs targeting primary school students, thereby promoting the optimization of SEC cultivation practices and policy improvements, ultimately fostering the holistic development and wellbeing of children.

## 2 Methods

The methodological design and implementation of this study strictly adhered to academic standards. The study protocol and systematic review process were pre-registered in the International Prospective Register of Systematic Reviews (PROSPERO, Registration number: CRD420251012368). Complete registration details can be accessed via the specified link (https://www.crd.york.ac.uk/PROSPERO). This study rigorously followed the Preferred Reporting Items for Systematic Reviews and Meta-Analyses (PRISMA) guidelines and implemented them with reference to the Practical Guide for Transparent Reporting of Systematic Reviews (Sarkis-Onofre et al., 2021; Page et al., 2021).

### 2.1 Search strategy

A comprehensive literature search was conducted across four databases: Web of Science, PubMed, EBSCO, and CNKI. English search terms included: “Exercise”, “Physical exercises”, “Training”, “Exercise Trainings”, “Physical Activity”, “social-emotional competence”, “emotional regulation”, “task performance”, “collaboration”, “open-mindedness”, “engaging with others”, “Adolescents”, “Teenager”, “children”, “students”, “primary school students”, “RCT”. Chinese search terms included: “体育活动”, “体育锻炼”, “体育教学”, “体育运动”, “社会情感能力”, “任务能力”, “情绪调节能力”, “开放能力”, “协作能力”, “交往能力”, “小学生”, “青少年”, “儿童”, “随机对照实验”. To ensure transparency and reproducibility of the search process, the search strategy strictly followed these steps: First, core concepts (primary school students, physical activity, social-emotional competence, randomized controlled trials) were defined based on the PICOS framework. Subsequently, for each concept, a combination of subject headings (e.g., MeSH in PubMed) and free-text terms were utilized, and search strings were constructed using Boolean operators (synonyms within the same group were connected with “OR”, while different conceptual groups were connected with “AND”). Finally, this search strategy, after appropriate adaptation, was executed simultaneously across the Web of Science, EBSCO, and CNKI databases. Two researchers performed the literature search independently, followed by a combined verification. The search period spanned from the inception of each database to April 2025.

### 2.2 Inclusion and exclusion criteria

Based on the PRISMA statement, the PICOS framework (Participants, Interventions, Comparators, Outcomes, Study design) was used to assess study eligibility (Liberati et al., 2009). The inclusion criteria are detailed in Table 1.

**Table 1:** Inclusion and exclusion criteria.

**PICOS elements**	**Inclusion criteria**	**Exclusion criteria**
P	Primary school students aged 5-13 years	Non-primary school students or studies combining subjects with other age ranges
I	Any form of physical activity	Non-physical activities
C	Control group receiving regular activities	No control group
O	Social-emotional competence (task performance, emotional regulation, collaboration, open-mindedness, and engaging with others)	Outcomes not related to social-emotional competence (task performance, emotional regulation, collaboration, open-mindedness, and Engaging with others)or insufficient outcome data.
S	RCT and cRCT	non-RCTs and noncRCTs

### 2.3 Dependent variables

Implementing PA interventions for primary school students can yield multiple benefits, including improvements in SEC. This study focused on the five sub-dimensions of SEC as dependent variables: emotional regulation, task performance, engaging with others, collaboration, and open-mindedness.

### 2.4 Literature screening and data extraction

All studies retrieved from the online databases were imported into EndNote X9 reference management software. Screening was conducted according to the exclusion and inclusion criteria to identify relevant studies. The included literature was systematically managed to ensure organized documentation and efficient retrieval. Concurrently, descriptive data from the included studies were meticulously compiled using Microsoft Excel software. The compiled data encompassed the following key information: primary author(s), publication year, study design, sample size, intervention(s), intervention duration, relevant SEC outcomes (mean and standard deviation [SD] or mean and standard error [SE] at baseline and post-intervention, or the difference in mean and SD/SE), measurement instruments used, time points of measurement, and main findings.

### 2.5 Measurement tool characteristics

The included studies employed diverse assessment tools for measuring SEC. To systematically evaluate their psychometric properties, a “Measurement Tool Characteristics Table” was constructed (see Table 2), covering each tool's reliability (Cronbach's α), validity evidence, applicable age range, cultural adaptability, and sensitivity for special needs children (such as Autism Spectrum Disorder and Attention-Deficit Hyperactivity Disorder). The summary indicates that most tools demonstrated good internal consistency (Cronbach's α > 0.70) and construct validity. However, some tools showed limitations in their applicability for special needs children and insufficient cross-cultural adaptability.

**Table 2:** Measurement tool characteristics.

**References**	**Social-emotional competence dimension**	**Primary measurement tool (abbreviation)**	**Reliability (Cronbach's α)**	**Validity**	**Applicable age range**	**Cultural adaptability**	**Sensitivity for special needs children**
(Luna et al. 2021)	Social-emotional competence	Self-efficacy Inventory for Multiple Intelligences(IAMI)	0.75-0.79	Validated in a Spanish adolescent population, demonstrating good construct and content validity.	10-13 years	Standardized in the Spanish cultural context	Not assessed
(Condello et al. 2021)	Engaging with others collaboration	Multisource Assessment of Children's Social Competence Scale(MASCS)	>0.7	Good construct validity.	Primary school students	Available in Greek, among other languages, and successfully used in Italian samples, demonstrating cross-cultural applicability	Not assessed
(Roh et al. 2018)	Emotional regulation	Profile of Mood States-Brief (POMS-B)	0.91	Good construct validity.	Adolescents & adults	Adapted and validated in Korean culture	Not assessed
(Roh et al. 2018)	Engaging with others	Sociability measurement model for juveniles	Good reliability	Good content and construct validity in the Korean adolescent population.	Children & adolescents	Locally developed in Korea, based on Korean cultural context and educational environment; excellent cultural adaptability	Not assessed
(Haghighi et al. 2023)	Engaging with others	Gilliam Autism Rating Scale-Second Edition(GARS-2)	>0.90	High criterion-related validity with the ABC scale (r > 0.75).	3–22years	Multiple country versions; excellent cultural adaptability	ASD
(Tse 2020)	Emotional regulation	Emotion Regulation Checklist(ERC)	0.7	Significant predictive validity.	6-12years	o apparent culture-specific content	Not assessed
	Engaging with others	Autism treatment evaluation checklist (ATEC)	>0.90	Good validity.	2-12years	Good cultural adaptability.	ASD.
(Zhao and Chen 2018a)	Engaging with others Collaboration	Social Skills Improvement System Rating Scales(SSIS-RS)	>0.90	High concurrent validity and good construct validity.	3-18years	Good cultural adaptability; used a Chinese version validated for reliability and validity.	ASD.
		Assessment of Basic Language and Learning Skills-Revised(ABLLS-R)	>0.90	Good content validity.	0-12 years	Cultural adaptability unclear. A Chinese translated version was used in this study, but systematic validation in the Chinese ASD population is lacking.	ASD.
Ángel Latorre-Román et al. (2021)	Open-mindedness	Torrance Tests of Creative Thinking (TTCT)	0.71–0.85	Significant predictive validity.	Kindergarten to adult	Applicable across multiple countries.	Not assessed.
(Williams et al. 2019)	Emotional Regulation	Children's Depression Inventory (CDI)	0.80–0.86	Good discriminative validity.	7-17 years	Multicultural norms available; attention to item comprehension required.	Not assessed
	Emotional Regulation	*Pediatric Anger Expression Scale (PAES)*	0.67–0.74	Good construct validity.	Children	Primarily validated in European and American samples	Not assessed.
(Bollimbala et al. 2019)	Open-mindedness	Guildford Alternate Uses (GAU)	>0.70	Construct and predictive validity supported by multiple studies.	11-13 years	Cross-culturally universal.	Not assessed.
	Open-mindedness	*Remote Association Test (RAT)*	0.85-0.87	Good construct validity.	11-13 years	Language-sensitive; requires systematic adaptation.	Not assessed
(Ludyga et al. 2022)	Task performance	Change detection task	Good theoretical construct validity	Good validity.	Primary school students	Cross-culturally universal.	ADHD.
(Zhou 2023)	Social-emotional competence	Children's social-emotional competence scale	>0.9	Good theoretical construct validity.	6-12 years	Developed based on the Chinese educational context, demonstrating good cultural adaptability and localized validity.	Not assessed

### 2.6 Statistical methods

Since multiple effect sizes were extracted from individual studies, a three-level meta-analysis was conducted using the metafor package in R version 4.5.1. For continuous outcome data where the direction of effect was reversed (i.e., in some studies, higher scores indicated a better outcome, while in others, lower scores indicated a better outcome), a reversal method was applied: the means were reversed before the results were pooled. For studies reporting both baseline and post-intervention data, effect sizes were calculated based on change scores (post-intervention minus baseline). When the correlation coefficient between baseline and post-intervention measurements was not reported for change score calculations, a coefficient of r = 0.5 was assumed based on recommendations from prior research (r = 0.5).

The three-level meta-analytic model partitioned the total variance into three components: sampling variance (Level 1), within-study variance (Level 2), and between-study variance (Level 3) (Cheung, 2014). Effect sizes are reported as Cohen's d values, along with their 95% confidence intervals (CI) and prediction intervals (PI). According to Cohen's benchmarks, an effect size of d = 0.20 is considered small, d = 0.50 is medium, and d = 0.80 is large.

To examine the specific effects of PA on the sub-dimensions of SEC (Emotional Regulation, task performance, Engaging with Others, Collaboration, and Open-mindedness), these sub-dimensions were included as moderators in the aforementioned three-level model for subgroup analysis. Within this framework, the overall effect size represents the grand mean of all effect sizes, whereas the pooled effect size for each sub-dimension is the weighted average of all effect sizes belonging to the same dimension under this model. This approach ensures that effect size estimates for different dimensions are based on the same statistical model, making the results comparable and effectively revealing the specific effects of PA on different SEC.

Publication bias was assessed using Stata version 18.0 software. The potential for publication bias was evaluated through funnel plots and tested using Egger's regression test (Egger et al., 1997).

## 3 Results

### 3.1 Search results

**Figure 1:**
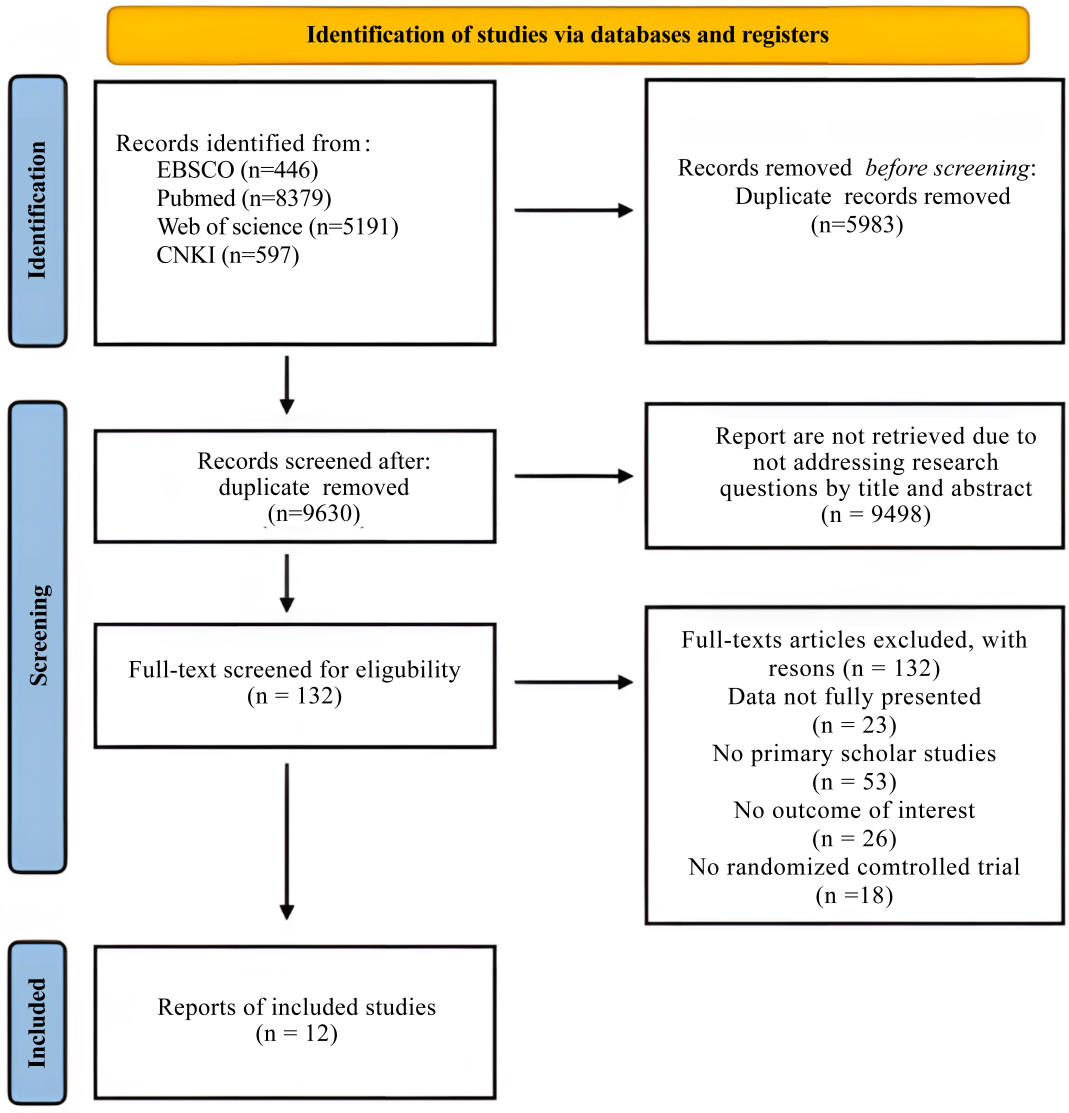
Flowchart of the study selection process according to the PRISMA protocol declarations.

A total of 15,486 articles were initially identified: 5,191 from Web of Science, 8,376 from PubMed, 1,293 from EBSCO, and 626 from CNKI (China National Knowledge Infrastructure). After importing these articles into EndNote software and removing duplicates, 9,503 articles remained. During title screening and abstract review, 9,371 articles were excluded as they did not meet the requirements, leaving 132 articles. Subsequent full-text review excluded 120 articles that did not meet the criteria related to outcome measures, data completeness, or participant characteristics. Ultimately, 12 studies were included, comprising 1 master's thesis and 11 journal articles. A detailed flowchart of the screening process is shown in Figure 1.

### 3.2 Basic information of included studies and intervention characteristics

All 12 included studies employed either randomized controlled trials (RCTs) or cluster randomized controlled trials (cRCTs) designs. Publication dates ranged from 1998 to 2023. Three studies were conducted in China, two in Spain, and two in Iran. The remainder (one each) were conducted in the United States, Italy, South Korea, India, and Switzerland.The total number of participants across all studies was 909. The sample size in the intervention groups ranged from 8 to 91 participants, and in the control groups from 8 to 90 participants. Six studies measured engaging with others, five measured emotional regulation, three measured task performance, three measured collaboration, and two measured open-mindedness. The basic characteristics of the included studies are summarized in Table 3.

**Table 3:** Characteristics of included studies.

**References**	**Age**		**Sample Size**		**Intervention**		**Intervention Duration**	**Outcomes**
	**Experimental Group**	**Control Group**	**Experimental Group**	**Control Group**	**Experimental Group**	**Control Group**		
(Luna et al. 2021)	10.76 ± 0.73		87	82	The sports game of the MooN program based on the sports education model	The sports game of the instructional unit based on the Traditional Model of Direct Instruction	9 weeks	Emotional regulation engaging with others
(Condello et al. 2021)	10.73 ± 0.32	10.72 ± 0.31	91	90	Structured physical activity	Regular physical activities	24 weeks	Collaboration
(Roh et al. 2018)	11.73 ± 0.64	11.40 ± 0.63	15	15	Taekwondo training	Regular physical activities	16 weeks	Emotional regulation
(Zhou 2023)	Aged 6-8 years		32	32	Open-ended physical activity games program	Traditional physical activity games program	11 weeks	Task performance collaboration engaging with others
(Ludyga et al. 2022)	10.8 ± 1.2	10 ± 1.2	28	28	Judo training	Routine daily activities	12 weeks	Task performance
(Bollimbala et al. 2019)	12 ± 1.29		17	17	Folk dance	Sedentary sitting for 20 mins, allowing social interaction but no physical activity.	20 mins	open-mindedness
(Haghighi et al. 2023)	9.00 ± 1.31	8.13 ± 1.36	8	8	Combined physical training	Routine daily activities	8 weeks	Engaging with others
(Zhao and Chen 2018a)	6.14 ± 0.96	6.1 ± 0.98	20	20	Structured physical activity	Regular physical activities	12 weeks	Collaboration engaging with others emotional regulation emotional regulation
Ángel Latorre-Román et al. (2021)	9.75 ± 1.12	9.94 ± 1.13	48	48	Structured physical activity	Non-participation in physical activity programs	20 mins	open-mindedness
(Najafabadi et al. 2018)	7.08 ± 2.06	8.07 ± 2.23	87	82	Structured physical activity	Regular physical activities	12 weeks	Engaging with others
(Tse 2020)	10.07 ± 1.10	9.42 ± 0.90	12	12	Aerobic exercise	Routine daily activities	12 weeks	Emotional regulation
(Williams et al. 2019)	9.7 ± 0.88	9.6 ± 0.88	85	85	Aerobic exercise	Sedentary behavior	8 months	Emotional regulation

### 3.3 Quality assessment of included studies

**Figure 2:**
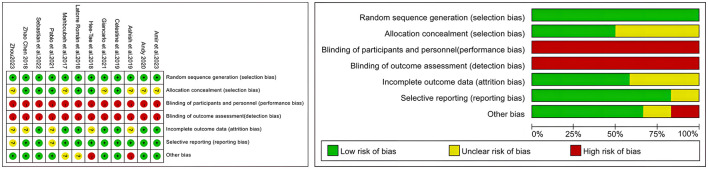
Quality assessment of the included studies.

Study quality was assessed using the Cochrane Risk of Bias tool, evaluating the following domains: random sequence generation, allocation concealment, blinding of participants and personnel, blinding of outcome assessment, incomplete outcome data, selective outcome reporting, and other sources of bias. As recommended, each criterion was rated as follows: “Low risk” if the study fully met quality standards with minimal bias; “Unclear risk” if bias concerns potentially affected result credibility; and “High risk” if bias seriously undermined result credibility. Disagreements between reviewers were resolved through consultation with other team members to reach a consensus-based judgment.Among the 12 RCT and cRCT included in the analysis, all studies adequately reported the random sequence generation and experimental group allocation procedures. However, methodological limitations existed in the operational details of allocation concealment. Due to the dual constraints of ensuring participant informed consent and the necessity for real-time medical supervision during exercise interventions, these studies faced inherent obstacles to implementing strict blinding during the trial phase.Based on the Cochrane Risk of Bias assessment, all studies were rated as “High risk” for selection bias. See Figure 2.

### 3.4 Physical activity interventions

The PA interventions implemented across the 12 included studies varied in type: Taekwondo (*n* = 1), physical games (*n* = 2), dance (*n* = 1), aerobic exercise (*n* = 2), physical training (*n* = 1), structured physical activity (*n* = 4), and Judo (*n* = 1).

### 3.5 Social-emotional competence

#### 3.5.1 Overall effect size

**Figure 3:**
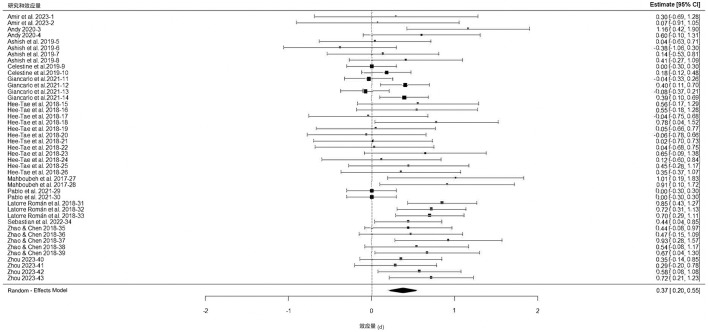
Forest plot of the meta-analysis on the intervention effects of physical activity on social-emotional competence in primary school students.

A three-level meta-analysis was conducted to systematically examine the effectiveness of PA interventions on the development of SEC in primary school students. Integrating data from the 12 studies yielded a total of 43 effect sizes (see Figure 3). In the variance equation, the correlation coefficient (rho) between pre- and post-intervention values needed specification. As none of the included published studies reported this value, rho = 0.5 was set as the substitute value for the meta-analysis. The results indicated a significant overall effect of PA interventions on SEC [*d* = 0.37, 95% *CI* (0.20, 0.55), *p* < 0.001]. According to Cohen's classification criteria, this d value represents a small-to-moderate effect size (Cohen, 1988). The 95% confidence interval was entirely above zero, indicating statistical significance. To assess overall variance heterogeneity, a Q-test was performed. The Q value for the three-level meta-analysis model was 67.662 (*p* = 0.007), indicating significant heterogeneity. Decomposition of heterogeneity revealed that variability among effect sizes within studies (Level 2) accounted for 14.79%, variability between studies (Level 3) accounted for 14.79%, and sampling error (Level 1) accounted for 70.42%. Within-study and between-study heterogeneity contributed approximately equally to the systematic variation. Although the degree of heterogeneity was low, the significant Q-test suggested the necessity of moderator analysis to further understand the specific mechanisms through which PA affects children's SEC.

#### 3.5.2 Publication bias risk assessment

**Figure 4:**
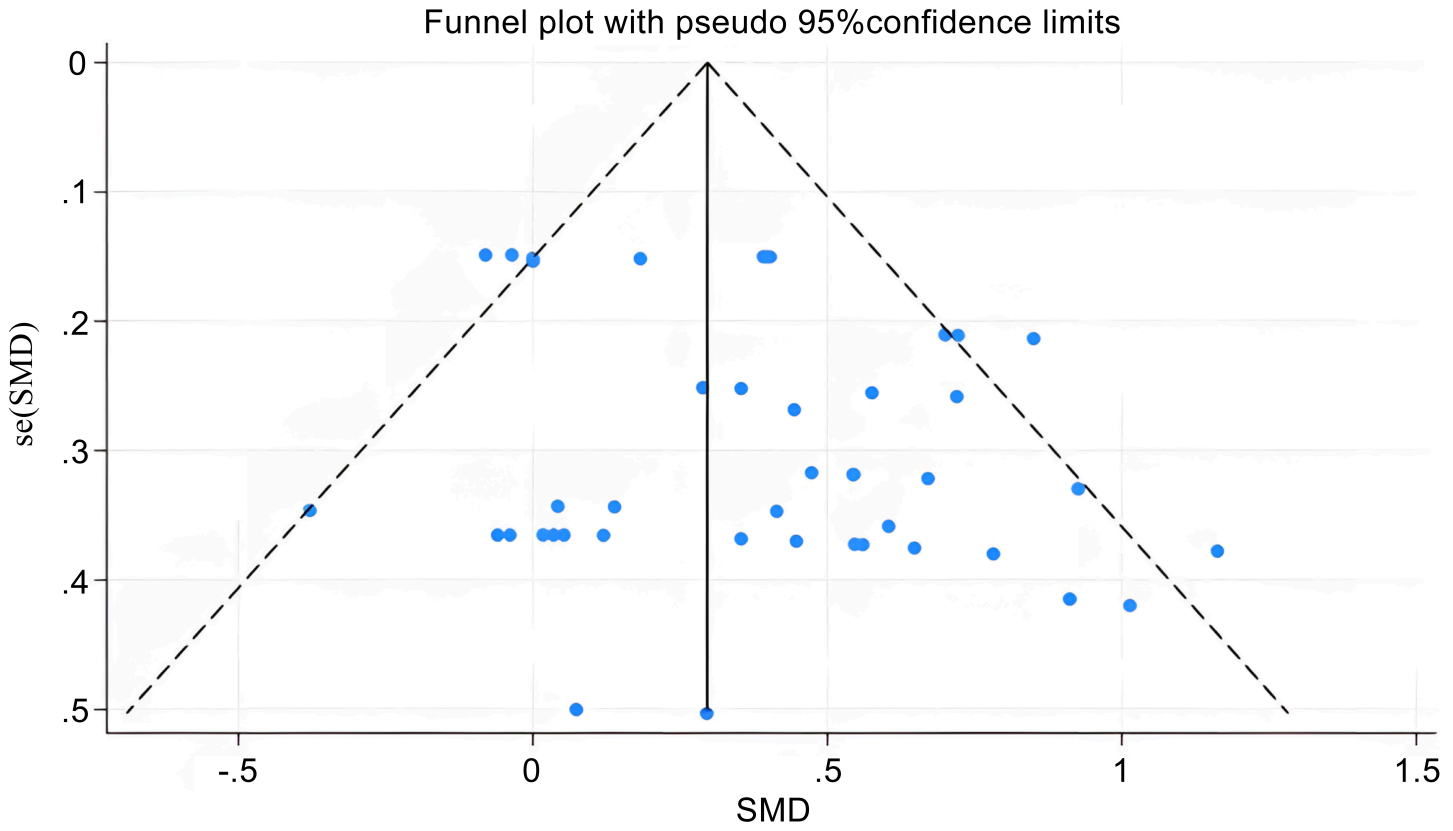
Funnel plot for publication bias assessment.

Publication bias was systematically assessed using a funnel plot complemented by Egger's regression test. As shown in Figure 4, the funnel plot exhibited some asymmetry, making it difficult to definitively assess publication bias based solely on visual inspection. Therefore, Egger's test was performed for quantitative analysis. The result of Egger's test was not statistically significant (*P* = 0.07, >0.05), suggesting a low risk of substantial publication bias among the included studies.

#### 3.5.3 Sensitivity analysis

To investigate potential sources of heterogeneity, a sensitivity analysis was performed by iteratively removing each individual effect size and then each individual study. The results demonstrated that after iteratively removing the 43 effect sizes, the pooled effect size fluctuated within the range of d = 0.351-0.386 (original *d* = 0.37), and all 95% confidence intervals excluded zero. Study-level sensitivity analysis showed that after excluding any single study, the pooled effect size fluctuated between *d* = 0.326-0.413, remaining consistent with the initial analysis and confirming the robustness of the overall conclusion. Considering that the “Open-mindedness” subgroup contained only 2 studies with a relatively small sample size, the relevant studies for this subgroup were excluded. The analysis showed that after exclusion, the effect size was *d* = 0.361 (95% CI: 0.183-0.539), essentially consistent with the full-sample results.

### 3.6 Dependent variables

This study revealed differential effect patterns across dimensions of SEC through systematic assessment of intervention outcomes. Collectively, the results revealed a small to medium effect size [*d* = 0.37, 95% CI (0.20, 0.55), *p* < 0.001], indicating that primary school students in the intervention group demonstrated significantly greater improvement in SEC relative to the control group. Data analysis indicated that all SEC sub-dimensions showed positive effects. Engaging with Others [*d* = 0.39, 95% CI (0.15, 0.63), *p* < 0.01] and Emotional Regulation [*d* = 0.36, 95% CI (0.08, 0.63), *p* = 0.01] reached statistically significant effects. The effects for task performance [*d* = 0.37, 95% CI (-0.03, 0.77), *p* = 0.07], Collaboration [*d* = 0.31, 95% CI (-0.01, 0.63), *p* = 0.06], and Open-mindedness [d = 0.43, 95% CI (-0.01, 0.87), p = 0.06] were marginally significant. Complete data distributions are detailed in Table 4.

**Table 4:** Effect sizes of the dependent variables.

**Dependent variable**	***n***	**Effect size(Cohen's d)**	***p***	**95%CI**	
				**LL**	**UL**
Overall social-emotional competence	43	0.37	< 0.001	0.20	0.55
Emotional regulation	12	0.36	0.01	0.08	0.63
Engaging with others	14	0.39	< 0.01	0.15	0.63
Collaboration	6	0.31	0.06	−0.01	0.63
Task performance	3	0.37	0.07	−0.03	0.77
open-mindedness	7	0.43	0.06	−0.01	0.87

### 3.7 Moderator variables

This study systematically examined several potential moderator variables to investigate their influence on the primary outcome. Within the analytical framework, Type of PA, Participants, Length of Intervention, and Research Period were incorporated as core moderating variables. Statistical analyses confirmed significant moderating effects of the examined variables on the effect size: Type of PA (*p* < 0.05), Participant classification (*p* < 0.0001), Length of Intervention (p < 0.0001), and Research Period (*p* < 0.01) each demonstrated statistically significant moderation; importantly, subgroup analyses revealed distinct patterns of influence associated with these moderators (complete data distributions are provided Table 5).

**Table 5:** Effect sizes for subgroup analysis of moderator variables in the intervention program.

**Moderator Variables**	**n**	**I^2^(%)**	**p**	**Effect size(d)**	**95%CI**	
					**LL**	**UL**
Total number of studies			< 0.01	0.37	0.20	0.55
Type of physical activity		14.10	< 0.05			
Physical games	6		0.36	0.24	−0.27	0.75
Dance	4		0.89	0.05	−0.71	0.81
Aerobic exercise	4		0.14	0.41	−0.14	0.96
Physical training	2		0.71	0.18	−0.79	1.15
Structured physical activity	14		< 0.01	0.58	0.20	0.95
Taekwondo	12		0.44	0.28	−0.43	0.99
Judo	1		0.28	0.44	−0.36	1.24
Length of Intervention		45.49	< 0.0001			
>12weeks	23		< 0.001	0.53	0.38	0.70
>12weeks	20		0.10	0.14	−0.03	0.31
Participants		26.27	< 0.0001			
Typically developing children	31		< 0.01	0.26	0.08	0.44
Children with special needs	12		< 0.001	0.62	0.34	0.90
Research Period		15.98	< 0.01			
Pre-pandemic	34		< 0.01	0.42	0.20	0.65
Post-pandemic	6		0.12	0.39	−0.11	0.89

#### 3.7.1 Type of physical activity

In subgroup analyses of Type of PA. intervention type moderated the relationship between PA and SEC in primary school students. Compared with other type of PA, structured physical activities demonstrated a significantly higher effect magnitude [*d* = 0.58, 95% CI (0.20, 0.95), *p* < 0.01].

#### 3.7.2 Participants

Participants were categorized into two subgroups: typically developing children and children with special needs, the latter explicitly defined as children with Autism Spectrum Disorder (ASD) or ADHD at the primary school level. The analysis demonstrated that the children with special needs subgroup exhibited a significantly stronger effect magnitude [*d* = 0.62, 95% CI (0.34, 0.90), *P* < 0.001] in response to PA intervention, compared with the typically developing children subgroup [d = 0.26, 95% CI (0.08, 0.44), *P* < 0.01].

#### 3.7.3 Length of intervention

To examine the moderating role of length of intervention, this study categorized interventions into shorter length ( ≤ 12 weeks) and longer length (>12 weeks). Compared with longer-length interventions [*d* = 0.14, 95% CI (-0.03, 0.31), *p* = 0.1], shorter-length interventions demonstrated a higher effect magnitude [*d* = 0.53, 95% CI (0.38, 0.70), *p* < 0.001]. This indicates that PA interventions of shorter length ( ≤ 12 weeks) may more effectively enhance SEC in primary school students, with statistical significance.

#### 3.7.4 Research period

To explore differences in the effects of physical interventions before and after the COVID-19 pandemic, the included studies were divided into two groups based on their implementation time: “Pre-pandemic” (studies implemented in 2019 and earlier) and “Post-pandemic” (studies implemented in 2020 and later). A total of 40 effect sizes were included (34 pre-pandemic, 6 post-pandemic). The three-level meta-analysis showed that physical interventions implemented before the pandemic had a significant positive effect on SEC [*d* = 0.42, 95% CI (0.20, 0.65), *P* < 0.01]. Interventions implemented after the pandemic also showed a positive trend but did not reach statistical significance [*d* = 0.39, 95% CI (-0.11, 0.89), *P* = 0.12]. The test for moderating effect indicated a significant difference between the two groups (QM = 15.98, *p* = 0.0003), but the smaller sample size of post-pandemic studies (n = 6) may have led to wider confidence intervals. In terms of point estimates, physical interventions both before and after the pandemic showed moderately positive effects.

## 4 Discussion

This meta-analysis included 12 studies with participants comprising primary school students aged 5 to 13 years. The primary objective was to quantitatively synthesize the effectiveness of PA interventions on the SEC of primary school students and systematically evaluate the intervention effects across the various dimensions of SEC. This was achieved by synthesizing diverse PA intervention programs targeting different populations from the literature, while also exploring the influence of three potential moderator variables: type of PA, participants, length of intervention, and research period.

### 4.1 Intervention effects on overall SEC and its sub-dimensions

The results of this study indicate that the pooled effect size for all PA interventions aimed at developing SEC in primary school students was statistically significant. The overall effect size for SEC pre- to post-intervention ranged from small to medium, suggesting the potential practical value of such interventions. This finding aligns with previous research. For instance, Wright et al. found that PA contributes to the promotion of SEC characterized by respect, participation, engagement, and cooperation (Wright et al., 2021). Based on the Comprehensive School Physical Activity Program (CSPAP) framework, Moon et al. conducted a meta-analysis of 27 studies and found a small-to-medium positive effect (Hedges' g = 0.44) of PA interventions on the SEC of primary school students (Moon et al., 2024). Another systematic review of meta-analyses also indicated that well-designed and well-implemented Social and Emotional Learning programs are associated with positive social, emotional, behavioral, and academic outcomes in children and adolescents, demonstrating a medium effect size (Jones and Bouffard, 2012).

The effects of PA interventions varied significantly across the different dimensions of SEC. Specifically, Engaging with Others and Emotional Regulation demonstrated significant medium effect sizes. For Engaging with Others, the social nature of PA provides primary school students with a platform to practice collaboration, empathy, and conflict resolution (Gano-overway et al., 2009). This can be achieved through diverse activity formats, guided approaches, and attention to students' psychological states. Students learn to interact with peers, respect rules, and cooperate with others, practicing collaborative and communication skills while interacting during shared task completion (Smith, 2003). Regarding Emotional Regulation, the promoting effect of PA is reflected in the synergistic mechanisms of physiological, psychological, and behavioral levels. From a neurophysiological perspective, moderate-intensity exercise promotes the release of endorphins, dopamine, and serotonin. Endorphins have natural analgesic and pleasurable effects, dopamine is involved in regulating the reward mechanism, and increased serotonin levels help stabilize mood (Basso and Suzuki, 2016). This improvement in the neurochemical environment lays the physiological foundation for emotional self-regulation. At the motivational and affective levels, the fun and challenge inherent in PA can effectively stimulate children's intrinsic motivation for communication (Weiss and Stuntz, 2004). Meanwhile, its inherent rule constraints, goal orientation, and immediate feedback characteristics provide children with real behavioral contexts to practice emotional management, allowing them to continuously learn how to regulate their own emotions in social interactions during exercise (Tse, 2020). The results indicated that the improvements in task performance, collaboration, and open-mindedness did not reach statistical significance. This finding requires careful interpretation in light of systematic measurement error inherent in the assessment tools. As detailed in Table 2 (Characteristics of Measurement Tools), the instruments used for these non-significant dimensions generally exhibited limitations in scale sensitivity and ecological validity, which may have indirectly increased the risk of bias from selective reporting of non-significant outcomes.

### 4.2 Effects of moderator variables

This study further examined potential moderators underlying the heterogeneity in PA interventions' effects on SEC, with a systematic focus on three key variables. The analysis identified Type of PA, Participants, Length of Intervention, and Research Period as significant moderators influencing intervention outcomes.

Regarding the type of PA, the significant effectiveness of structured physical activity may be related to its goal-oriented design (Wright et al., 2021). Structured physical activity is an organized, planned form of activity with clear objectives and a specific structure. It achieves intervention effects through mechanisms such as behavioral reinforcement (e.g., immediate feedback rewarding cooperative behavior) and situational simulation (e.g., recreating real social conflicts within sports scenarios) (Zhao and Chen, 2018b). This integrated model transforms social-emotional learning from passive reception into active practice, forming a sustainable pathway for competency development, thereby enhancing the SEC of primary school students. Furthermore, structured physical activity can enhance neural activity in the prefrontal and parietal lobes, optimizing the executive control network (e.g., inhibiting distractions, flexibly switching strategies) and improving performance in complex tasks, consequently boosting SEC (Hillman et al., 2014).

Concerning participant characteristics, ASD commonly exhibit sensory processing disorders and low social competence. PA provides them with opportunities to interact with others. Simultaneously, targeted PA (e.g., tactile games, proprioceptive training) can help integrate multisensory signals in the brain by providing organized sensory stimulation, effectively alleviating anxiety and reducing repetitive behaviors (Sowa and Meulenbroek, 2012; Bass et al., 2009). Previous research has shown that appropriate regular exercise interventions can effectively improve issues such as flexibility, cognitive control, social skills, behavioral problems, motor skills, and coordination in children with ASD (Wang et al., 2025; Koh, 2024). Children with ADHD often have delayed development of the prefrontal cortex. Exercise improves inhibitory control and attention by increasing dopamine and norepinephrine secretion (Williams and Thayer, 2009).

Regarding Length of intervention, results demonstrated that shorter-length interventions ( ≤ 12 weeks) yielded significantly greater improvements in SEC compared to longer-length interventions (>12 weeks). This aligns with prior research indicating that brief, intensive interventions effectively promote positive outcomes in children and adolescents‘ social-emotional development (Gutman and Schoon, 2015). The observed superiority of shorter interventions may arise from their enhanced capacity to deliver immediate performance feedback, introduce optimally challenging novel tasks that provide novelty, and implement autonomy-supportive structures; these mechanisms collectively strengthen Participants' sense of competence and autonomy, thereby activating intrinsic motivation to drive behavioral changes that facilitate social-emotional growth (Gutman and Schoon, 2015). Concurrently, the reduced risk of boredom and burnout associated with shorter Length of intervention positively influences intervention efficacy (Yeager, 2017). However, the finding that “shorter interventions are more effective” requires cautious interpretation, and alternative explanations should be fully considered. First, there may be measurement timing bias: most studies only conducted post-tests immediately after the intervention, capturing immediate effects rather than long-term impacts (Durlak, 2020). Second, the sustainability of intervention effects warrants attention. Relying solely on immediate post-testing may overestimate long-term benefits, as school-based SEC interventions often show significant decay in follow-up assessments (Sklad et al., 2012).

Notably, our subgroup analysis based on the research period revealed that although the effect size of PA interventions implemented after the pandemic did not reach statistical significance, the point estimate (*d* = 0.39) still demonstrated a moderate positive trend. This suggests that PA retains meaningful intervention potential in the post-pandemic context. This finding carries important real-world relevance. A large-sample repeated cross-sectional study conducted by (Yang et al. 2025) in Ningbo showed that after the adjustment of dynamic zero-COVID policies, the detection rate of depressive symptoms among adolescents increased from 14.6% to 17.0%, and the rate of school bullying victimization rose from 1.7% to 3.2%, nearly doubling (Yang et al., 2025). These data clearly illustrate the severe challenges adolescents face in emotional regulation and social interaction in the post-pandemic era (Liu et al., 2025). Based on these findings, to better address children's psychosocial development needs, we recommend that schools prioritize “structured” instructional design in physical education curricula, systematically integrate social-emotional learning objectives into the content, and employ short-cycle, well-targeted intervention strategies to specifically enhance students' emotional regulation and interpersonal skills (Luna et al., 2021).

## 5 Limitations

This study has several limitations. First, although a comprehensive and systematic literature search strategy was employed, it is possible that studies using different keyword formulations or published in other languages were not captured, which may have affected the breadth of literature coverage. Future efforts should focus on developing a multidimensional keyword database by compiling comprehensive synonyms and cross-domain expressions of core variables, supported by machine translation tools to enhance cross-language retrieval and significantly improve search coverage. Second, only 12 studies met the eligibility criteria for this review. Although numerous studies have used PA as an intervention, relatively few directly targeted and reported outcomes related to SEC in primary school students, which somewhat limits the generalizability of the findings. Future research should encourage more large-sample, multi-center randomized controlled trials with SEC as a primary endpoint in this population, alongside promoting open data sharing to establish a field-specific raw data platform and accumulate richer original data to support conclusions. Third, the current findings primarily support the efficacy of PA interventions in enhancing SEC among primary school students. Whether similar effects exist for other age groups remains insufficiently evidenced. Subsequent work should extend interventions across the lifespan, comparing effect differences among various age groups to precisely identify critical developmental windows and inform age-stratified physical education policies. Fourth, there are methodological limitations in measurement approaches. Most existing studies rely on traditional self-report or observer-rated scales, which are susceptible to recall bias, social desirability effects, and rater subjectivity, thereby constraining measurement objectivity. Future studies could adopt emerging technological assessment tools—such as VR-based scenario assessments and tablet-based gamified tools (e.g., NIH Baby Toolbox)—and integrate multimodal data combining behavioral observations and physiological indicators to enhance assessment precision and more accurately elucidate the mechanisms underlying PA effects.

## 6 Conclusions

This meta-analysis confirms that PA can effectively promote the development of SEC among primary school students, with particularly significant improvements observed in engaging with others and emotional regulation. Subgroup analyses further revealed distinct effect variations across intervention approaches: structured physical activities and shorter-length interventions ( ≤ 12 weeks) generated positive impacts for both typically developing children and those with special needs, with especially pronounced benefits observed in the special needs population.

To support the implementation of the “Double Reduction” policy and advance the “Five Educations Simultaneously” educational framework, we recommend establishing a multi-tiered PA intervention system. This system should incorporate a “structured curriculum + sports clubs” model for general students, delivering physical activities three times weekly in 40 min sessions. For students with special needs, individualized programs should be developed following a “personalized prescription” approach—for instance, designing sessions that alternate between aerobic exercise and team games for children with ADHD, using 60%-75% of maximum heart rate as the moderate-intensity threshold. Additionally, we recommend introducing qualified community sports resources such as fencing and rock climbing through a “government-recommended + school-autonomous” mechanism to diversify program content and enhance student engagement. Finally, PA outcomes should be integrated into school mental health assessment systems, with regional intervention databases established to continuously monitor and optimize implementation strategies, thereby maximizing the role of physical education in promoting holistic student development.

## Data Availability

The data analyzed in this study is subject to the following licenses/restrictions: The original contributions presented in the study are included in the article/Supplementary material, further inquiries can be directed to the corresponding author. Requests to access these datasets should be directed to Simei Fu, 2323936454@qq.com.
