# Supplementary material for: The effects of physical activity on social-emotional competence in primary school students: a meta-analysis
Source: Front Psychol. 2025 Nov 13;16:1657165. doi: 10.3389/fpsyg.2025.1657165 (PMC12659682; doi:10.3389/fpsyg.2025.1657165)
Supplement: Supplementary file 1 [file Data_Sheet_1.pdf]

| NO. | PubMed Search terms |
|---|---|
| #1 | “Exercises”[MeSH Terms] OR “Exercise, Physical”[Title/Abstract] OR “Exercises, Physical”[Title/Abstract] OR “Physical Exercise”[Title/Abstract] OR “Physical Exercises”[Title/Abstract] OR“Exercise, Aerobic”[Title/Abstract] OR“Aerobic Exercise”[Title/Abstract] OR“Aerobic Exercises”[Title/Abstract] OR“Exercises, Aerobic”[Title/Abstract] OR“Exercise, Isometric”[Title/Abstract] OR“Exercises, Isometric”[Title/Abstract] OR“Isometric Exercises”[Title/Abstract] OR “Isometric Exercise”[Title/Abstract] OR“Acute Exercise”[Title/Abstract] OR“Acute Exercises”[Title/Abstract] OR“Exercise, Acute”[Title/Abstract] OR “Exercises, Acute”[Title/Abstract] OR“Exercise Training”[Title/Abstract] OR“Exercise Trainings”[Title/Abstract] OR“Training, Exercise”[Title/Abstract] OR“Trainings, Exercise”[Title/Abstract] OR“Physical Activity”[Title/Abstract] OR“Activities, Physical”[Title/Abstract] OR“Activity, Physical”[Title/Abstract] OR“Physical Activities”[Title/Abstract] |
| #2 | “social emotional competence”[Title/Abstract] OR “social emotional learning”[Title/Abstract] OR “social emotional skills”[Title/Abstract] OR “social emotional development”[Title/Abstract] OR “social-emotional learning”[Title/Abstract] OR “Emotional Regulation”[MeSH Terms] OR “Emotional Regulations”[Title/Abstract] OR “Regulation, Emotional”[Title/Abstract] OR “Regulations, Emotional”[Title/Abstract]l OR “Emotion Regulation”[Title/Abstract] OR “Regulation, Emotion”[Title/Abstract] OR “Emotional Self-Regulation”[Title/Abstract] OR “Emotional Self Regulation”[Title/Abstract] OR “Emotional Self-Regulations”[Title/Abstract] OR “Self-Regulation, Emotional”[Title/Abstract] OR “Self-Regulations, Emotional”[Title/Abstract] OR “Emotion Self-Regulation”[Title/Abstract] OR “Emotion Self Regulation”[Title/Abstract] OR “Emotion Self-Regulations”[Title/Abstract] OR “Self-Regulation, Emotion”[Title/Abstract] OR “Self-Regulations, Emotion”[Title/Abstract] OR “Task Competence”[Title/Abstract] OR “Task Performance”[Title/Abstract] OR “Task Management Skills”[Title/Abstract] OR “Task Execution Ability”[Title/Abstract] OR “Task Proficiency”[Title/Abstract] OR “Task-Oriented Skills”[Title/Abstract] OR “Task Completion Ability”[Title/Abstract] OR “Task Planning and Execution”[Title/Abstract] OR “Self-Regulation in Tasks”[Title/Abstract] OR “Goal-Directed Behavior”[Title/Abstract] OR “Executive Function”[Title/Abstract] OR “Cognitive Skill”[Title/Abstract]s OR “Openness Ability”[Title/Abstract] OR “Open-mindedness”[Title/Abstract] OR “Willingness to Try New Things”[Title/Abstract] OR “Adaptability”[Title/Abstract] OR “Flexibility in Social Interactions”[Title/Abstract] OR “Innovational Thinking”[Title/Abstract] OR “Creative Thinking”[Title/Abstract] OR “Innovative Thinking”[Title/Abstract] OR “Curiosity OR Inquisitiveness”[Title/Abstract] OR “Interest OR Inclusiveness”[Title/Abstract] OR “Tolerance OR |

| | |
|---|---|
| | Acceptance"[Title/Abstract] OR "Open-mindedness OR Embrace"[Title/Abstract] OR "Interpersonal Relation"[Title/Abstract] OR "Relation, Interpersonal"[Title/Abstract] OR "Social Relationships"[Title/Abstract] OR "Relationship, Social"[Title/Abstract] OR "Social Relationship"[Title/Abstract] OR "Partner Communication"[Title/Abstract] OR "Communication, Partner"[Title/Abstract] OR "Partner Communications"[Title/Abstract] |
| #3 | "primary school students"[Title/Abstract] OR "child [MeSH Terms] OR "children"[Title/Abstract] OR "students"[Title/Abstract] OR "Adolescents"[Title/Abstract] OR "Adolescence"[Title/Abstract] OR" Adolescents, Female"[Title/Abstract] OR"Adolescent, Female"[Title/Abstract] OR Female Adolescent"[Title/Abstract] OR"Female Adolescents"[Title/Abstract] OR"Adolescents, Male"[Title/Abstract] OR"Adolescent, Male"[Title/Abstract] OR"Male Adolescent"[Title/Abstract] OR "Male Adolescents"[Title/Abstract] OR"Youth OR Youths"[Title/Abstract] OR"Teens"[Title/Abstract] OR"Teen"[Title/Abstract] OR"Teenagers"[Title/Abstract] OR"Teenage"[Title/Abstract] |
| #4 | "randomized controlled trial"[Publication Type] OR "randomised controlled trial"[Title/Abstract] OR "RCT"[Title/Abstract] OR "randomized"[Title/Abstract] OR"randomised"[Title/Abstract] OR "randomly"[Title/Abstract] OR"controlled trial"[Title/Abstract] OR"clinical trial"[Title/Abstract] |
| #5 | #1 AND #2 AND #3 AND #4 |

| NO. | Web of science Search terms |
|---|---|
| #1 | Exercises OR Exercise, Physical OR Exercises, Physical OR Physical Exercise OR Physical Exercises OR Exercise, Aerobic OR Aerobic Exercise OR Aerobic Exercises OR Exercises, Aerobic OR Exercise, Isometric OR Exercises, Isometric OR Isometric Exercises OR Isometric Exercise OR Acute Exercise OR Acute Exercises OR Exercise, Acute OR Exercises, Acute OR Exercise Training OR Exercise Trainings OR Training, Exercise OR Trainings, Exercise OR Physical Activity OR“Activities, Physical OR Activity, Physical OR Physical Activities |
| #2 | social emotional competence OR social emotional learning OR social emotional skills OR social emotional development OR social-emotional learning OR Emotional Regulation OR Emotional Regulations OR Regulation, Emotional OR Regulations, Emotional OR Emotion Regulation OR Regulation, Emotion OR Emotional Self-Regulation OR Emotional Self Regulation OR Emotional Self-Regulations OR Self-Regulation, Emotional OR Self-Regulations, Emotional OR Emotion Self-Regulation OR Emotion Self Regulation OR Emotion Self-Regulations OR Self-Regulation, Emotion OR Self-Regulations, Emotion OR Task Competence OR Task Performance OR Task Management Skills OR Task Execution Ability OR Task Proficiency OR Task-Oriented Skills OR Task Completion Ability OR Task Planning and Execution OR Self-Regulation in Tasks OR Goal-Directed Behavior OR Executive Function OR Cognitive Skills OR Openness Ability OR Open-mindedness OR Willingness to Try New Things OR Adaptability OR Flexibility in Social Interactions OR Innovational Thinking OR Creative Thinking OR Innovative Thinking OR Curiosity OR Inquisitiveness OR Interest OR Inclusiveness OR Tolerance OR Acceptance OR Open-mindedness OR Embrace OR Interpersonal Relation OR Relation, Interpersonal OR Social Relationships OR Relationship, Social OR Social Relationship OR Partner Communication OR Communication, Partner OR Partner Communications |
| #3 | primary school students OR child OR children OR students OR Adolescents OR Adolescence OR Adolescents, Female OR Adolescent, Female OR Female Adolescent OR“Female Adolescents OR Adolescents, Male OR Adolescent, Male OR Male Adolescent OR Male Adolescents OR Youth OR Youths OR Teens OR Teen OR Teenagers OR Teenage |
| #4 | randomized controlled trial OR randomised controlled trial OR RCT OR randomized OR randomised OR randomly OR controlled trial OR clinical trial |
| #5 | #1 AND #2 AND #3 AND #4 |

| NO. | EBSCO Search terms |
| --- | --- |
| #1 | Exercises OR Exercise, Physical OR Exercises, Physical OR Physical Exercise OR Physical Exercises OR Exercise, Aerobic OR Aerobic Exercise OR Aerobic Exercises OR Exercises, Aerobic OR Exercise, Isometric OR Exercises, Isometric OR Isometric Exercises OR Isometric Exercise OR Acute Exercise OR Acute Exercises OR Exercise, Acute OR Exercises, Acute OR Exercise Training OR Exercise Trainings OR Training, Exercise OR Trainings, Exercise OR Physical Activity OR"Activities, Physical OR Activity, Physical OR Physical Activities |
| #2 | social emotional competence OR social emotional learning OR social emotional skills OR social emotional development OR social-emotional learning OR Emotional Regulation OR Emotional Regulations OR Regulation, Emotional OR Regulations, Emotional OR Emotion Regulation OR Regulation, Emotion OR Emotional Self-Regulation OR Emotional Self Regulation OR Emotional Self-Regulations OR Self-Regulation, Emotional OR Self-Regulations, Emotional OR Emotion Self-Regulation OR Emotion Self Regulation OR Emotion Self-Regulations OR Self-Regulation, Emotion OR Self-Regulations, Emotion OR Task Competence OR Task Performance OR Task Management Skills OR Task Execution Ability OR Task Proficiency OR Task-Oriented Skills OR Task Completion Ability OR Task Planning and Execution OR Self-Regulation in Tasks OR Goal-Directed Behavior OR Executive Function OR Cognitive Skills OR Openness Ability OR Open-mindedness OR Willingness to Try New Things OR Adaptability OR Flexibility in Social Interactions OR Innovational Thinking OR Creative Thinking OR Innovative Thinking OR Curiosity OR Inquisitiveness OR Interest OR Inclusiveness OR Tolerance OR Acceptance OR Open-mindedness OR Embrace OR Interpersonal Relation OR Relation, Interpersonal OR Social Relationships OR Relationship, Social OR Social Relationship OR Partner Communication OR Communication, Partner OR Partner Communications |
| #3 | primary school students OR child OR children OR students OR Adolescents OR Adolescence OR Adolescents, Female OR Adolescent, Female OR Female Adolescent OR"Female Adolescents OR Adolescents, Male OR Adolescent, Male OR Male Adolescent OR Male Adolescents OR Youth OR Youths OR Teens OR Teen OR Teenagers OR Teenage |
| #4 | randomized controlled trial OR randomised controlled trial OR RCT OR randomized OR randomised OR randomly OR controlled trial OR clinical trial |
| #5 | #1 AND #2 AND #3 AND #4 |

| NO. | CNKI Search terms |
| --- | --- |
| #1 | 社会情感 + 社会情感能力 + 社会情感学习 + 社会情感发展 + 社会情感技能 + 社会情感教育 |
| #2 | 体育 + 体育教学 + 体育运动 + 体育活动 + 体育锻炼 + 体育课 |
| #3 | 小学生 |
| #4 | 随机 + 随机抽样 + 随机对照试验 + 随机对照研究 + 随机对照 |
| #5 | #1 AND #2 AND #3 AND #4 |
